# Impact of sperm DNA fragmentation index on assisted reproductive outcomes: a retrospective analysis

**DOI:** 10.3389/fendo.2024.1530972

**Published:** 2025-01-21

**Authors:** Bin Yang, Leizhen Xia, Rufei Deng, Liping Wu, Zhiqin Zhang, Xingwu Wu, Tao Ding, Yan Zhao, Jialyu Huang, Zhihui Huang

**Affiliations:** ^1^ Center for Reproductive Medicine, Jiangxi Branch of National Clinical Research Center for Obstetrics and Gynecology, Jiangxi Maternal and Child Health Hospital, Nanchang Medical College, Nanchang, China; ^2^ Medical Center of Burn Plastic and Wound Repair, The First Affiliated Hospital, Jiangxi Medical College, Nanchang University, Nanchang, China; ^3^ Bright Prosperity Research Center, Hangzhou, China

**Keywords:** sperm DNA fragmentation index, embryonic development, pregnancy outcomes, perinatal outcomes, neonatal complications

## Abstract

**Background:**

The role of sperm DNA fragmentation index (DFI) in fertility remains controversial. Herein, we analyzed its association with semen parameters, embryonic development, and pregnancy outcomes after *in vitro* fertilization (IVF) treatment. Additionally, we assessed whether DFI had a potential impact on long-term maternal and neonatal complications.

**Methods:**

A total of 5,271 women who underwent IVF treatment for the first time between October 1, 2020, and July 31, 2023, were included from an academic fertility center. Participants were categorized into three groups based on sperm DFI: DFI < 15%, 15 ≤ DFI < 30%, and DFI ≥ 30%. We collected data on patient demographics, semen parameters, embryonic development, clinical outcomes, maternal and infant complications. Multivariate logistic regression analyses were conducted to control for potential confounders.

**Results:**

The DFI value was negatively correlated with semen quality in males. High DFI affected the blastocyst formation rate (56.44%, 55.32%, 53.72%, respectively; P=0.045) and the rate of transferable embryos (3.97 ± 2.71, 3.90 ± 2.7, 3.38 ± 2.4, respectively; P<0.001); however, no significant difference in pregnancy outcomes was observed among the three groups. Elevated DFI did not contribute to clinically relevant adverse maternal events during pregnancy, but it was associated with an increased risk of low birth weight (3.9%, 6.6%, 10.1%, respectively; P=0.006) in newborns.

**Conclusions:**

Sperm DFI could influence embryonic development, with a higher risk of low birthweight infants in the high DFI group. However, it does not appear to affect clinical outcomes or other perinatal complications. The role of DFI as a predictive factor in assisted reproduction, especially regarding offspring outcomes, requires further investigation with larger sample sizes.

## Introduction

It is well known that male factors contribute to infertility at approximately the same rate as female factors (1, 2). However, the exact causes of male infertility remain poorly understood. While traditional semen analysis focusing on sperm concentration, motility and morphology, has been commonly used to assess male fertility, accumulating research suggests that these parameters do not always correlate with the outcomes of assisted reproductive technology (ART) (3). Recently, sperm DNA fragmentation (SDF) assessment has gained attention as a potential indicator of male fertility, as reduced sperm DNA integrity has been observed in infertile patients across various diagnostic assays (4, 5).

Sperm DNA is highly organized, and the degree of chromatin organization can influence epigenetic changes and embryo development (2, 6). The extent of sperm DNA damage is typically measured by the sperm DNA fragmentation index (DFI). However, there is ongoing debate regarding the impact of DFI on assisted reproductive outcomes. Some studies suggest that an increased DFI adversely affects both natural conception (7) and ART outcomes (8, 9). High DFI can even disrupt normal physiological functions, leading to the transmission of incorrect genetic information to offspring, which routine semen analysis cannot assess (5). Two meta-analyses have shown that elevated sperm DFI is associated with lower rates of good-quality embryos, reduced clinical pregnancy rates, and increased miscarriage rates (10, 11). Nonetheless, other meta-analyses have concluded that sperm DFI does not predict IVF or intracytoplasmic sperm injection (ICSI) outcomes (12). Based on the available literature, the impact of sperm DFI on embryonic development, clinical outcomes, and particularly on perinatal and neonatal outcomes, remains to be fully understood.

The American Urological Association (AUA) and the European Association of Urology (EAU) have acknowledged the importance of SDF in their 2023 guidelines on male infertility (13, 14). To establish a definitive correlation, a rigorous investigation with a large sample size and extended study duration is essential. In our retrospective study, we explored the effects of sperm DFI on embryonic development, clinical outcomes, and the risk of adverse maternal and neonatal outcomes in singleton pregnancies.

## Materials and methods

### Study design and participants

This retrospective cohort study enrolled a total of 5271 infertile couples who underwent IVF for the first time at the Reproductive Medicine Center of Jiangxi Maternal and Child Health Hospital, affiliated with Nanchang Medical College from October 1, 2020 to July 31, 2023.

The study was approved by the Reproductive Medicine Ethics Committee of Jiangxi Maternal and Child Health Hospital (Approval No. 2024136) and adhered to the principles outlined in the Declaration of Helsinki. All patients provided written informed consent for data collection and the anonymous use of their information in scientific research.

The following patients were included in this study: (1) women aged 20–40 years; (2) patients with infertility attributed to female pelvic cavity or tubal factors, or male factors, who met the indications for IVF/ICSI treatment; (3) an antral follicle count (AFC) ≥ 5, a basal follicle stimulating hormone level ≤ 10mIU/ml or an anti-mullerian hormone (AMH) level ≥ 1.2 ng/ml; (4) normal chromosomes, reproductive organs, and sexual function in both partners. The exclusion criteria were as follows: (1) natural cycle pregnancies; (2) women with stage III or IV endometriosis; (3) males with azoospermia or undergoing thawing or testicular/epididymal aspiration; (4) males with testicular atrophy, genital tract malformation or urogenital infection; and (5) either partner with chromosomal abnormalities or contraindications to assisted reproduction. Based on the DFI values, the finally enrolled male participants were categorized into three groups: DFI < 15%, 15% ≤ DFI < 30%, and DFI ≥ 30%.

### Semen collection and analysis

Semen samples were collected by masturbation after 2 to 7 days of ejaculatory abstinence, following the guidelines outlined in the Laboratory Manual of the WHO for the Examination and Processing of Human Semen (6th edition) (15). Each semen sample was collected into a sterile plastic cup and incubated at 37°C until complete liquefaction was achieved. The semen analysis, including the DFI test values, were collected within one month prior to IVF/ICSI procedures.

Routine semen analysis was performed using the SAS Differential Version Sperm Quality Analyzer (SAS - II, SAS Medical, China). Sperm concentration and viability were automatically recorded. For morphological evaluation, seminal smears were prepared and stained using the Diff - Quik staining method (Sperm Morphology Staining Kit, Anhui ANKE Biotechnology, China). Approximately 10 μL of semen was spread into a thin, homogeneous layer on a clean glass slide and air-dried at room temperature for at least 10 minutes. The slides were then stained and examined under a microscope (BX41; Olympus, Tokyo, Japan). According to WHO guidelines, sperm with deformed heads, midpieces, or principal pieces were classified as abnormal. The Sperm Deformity Index (SDI) was calculated as the number of deformed sperm divided by the total number of sperm. For each semen sample, at least 200 sperm were counted using a double-blind method. The percentage of sperm with normal morphology was then calculated.

### Sperm DFI assessment

Sperm DFI was assessed using the SCSA method (Sperm Nuclear Integrity Staining Kit, XingBo Biotechnology) on a flow cytometer (DxFlex, Beckman Coulter, USA), strictly in accordance with the product instructions (16). In this method, the chromatin in sperm nuclei with damaged DNA forms a single-stranded structure after acid treatment, which binds to acridine orange and emits red or yellow fluorescence. In contrast, chromatin in normal sperm nuclei retains a double-stranded structure after acid treatment and emits green fluorescence when bound to acridine orange. The proportion of sperm with damaged DNA is recorded as the DFI.

### Ovarian stimulation, embryo culture and fresh transfer

The female patients underwent a regular long gonadotropin - releasing hormone agonist (GnRH - α) regimen for controlled ovarian stimulation. The follicle development was monitored by transvaginal ultrasound and serum estradiol (E2) levels. Once the leading follicle size reached 20 mm or ≥ 3 follicles reached 18 mm, human chorionic gonadotropin (HCG) was injected intravascularly for maturation. Oocyte retrieval was performed 36 hours later under ultrasound guidance, followed by insemination using either conventional IVF or ICSI, depending on semen quality. For first-time patients, ICSI is recommended only in cases of severe male-factor infertility, defined as a sperm concentration < 5 million/mL, forward motility (type a+b) < 10%, or the presence of specific types of teratozoospermia (15, 17, 18). In all other cases, IVF is performed, with the decision remaining independent of DFI values. Fertilization was assessed 16 - 18 h after insemination as recommended by ESHRE (19). Day 3 embryos were assessed at 68 ± 1 h post-insemination, and those graded I-II with 7 - 10 blastomeres were high - quality embryos according to the Cummins’ criteria (20). Day 5 - Day 6 blastocysts were graded using the Gardner method (21), with score ≥ 4BB considered high - quality embryos.

Fresh embryo transfer was scheduled after progesterone transformation. Up to two embryos were transferred 3 or 5 days later depending on the developmental stage. Daily luteal phase support was provided with vaginal progesterone gel (90 mg/d, Crinone, Merck Serono) and oral dydrogesterone (20 mg/d, Duphaston, Abbott), and continued until 10 gestational weeks.

### Outcome parameters

For laboratory results, the oocyte retrieval rate was calculated by dividing the number of oocytes retrieved by the number of follicles ≥ 14 mm on trigger day. The ICSI mature oocytes (MII oocytes) rate and normal fertilization rate were determined by dividing the number of MII oocytes and 2 pronuclei (PN) zygotes by the total number of injected oocytes, respectively. The cleavage rate was obtained by dividing the number of day 3 cleavage-stage embryos produced from 2PN oocytes by the total number of 2PN oocytes. The good-quality embryo rate was calculated by dividing the number of good - quality embryos on day3 by the total number of embryos at the cleavage stage. The blastocyst formation rate was calculated by dividing the number of blastocysts by the number of day 3 embryos subjected to extended culture. Finally, the available blastocyst rate was calculated by dividing the number of available blastocysts by the total number of blastocysts on days 5 and 6.

Regarding pregnancy outcomes, biochemical pregnancy was defined as a serum Beta-human chorionic gonadotropin (β - hCG) level of ≥ 5 mIU/mL at 10–12 days after embryo transfer. Clinical pregnancy was determined by the identification of at least one gestational sac, with or without a fetal heartbeat, one month following transfer. The implantation rate was measured by dividing the number of gestational sacs by the number of embryos transferred. Miscarriage was defined as clinical pregnancy loss before the 24th gestational week, and live birth was defined as delivery of infants with viable signs after the 24th gestational week.

Obstetrical and neonatal outcomes were collected from couples by specially trained nurses using standardized questionnaires. Telephone surveys were conducted during each trimester of pregnancy and after delivery. Major obstetric complications assessed included hypertensive disorders of pregnancy (HDP), gestational diabetes mellitus (GDM), intrahepatic cholestasis of pregnancy (ICP), placenta previa, premature rupture of membranes, postpartum hemorrhage and cesarean delivery. Key neonatal outcomes included gender, gestational age, birthweight, Z - score, and major birth defects.

### Statistical analysis

Continuous variables were summarized as means with standard deviations and assessed for normality using the Shapiro - Wilk. Normally distributed data were compared using one-way analysis of variance (ANOVA), while skewed data were analyzed using the Kruskal-Wallis test. Categorical variables were presented as numbers and percentages, with comparisons were made using Pearson’s Chi - square test or Fisher’s exact test, as appropriate.

Multivariate logistic regression analyses were conducted to assess the independent effect of DFI. Adjusted variables included age, duration of infertility, type of infertility (primary or secondary), number of oocytes retrieved, endometrial thickness, presence of uterine scarring, number and type of embryos transferred, and fertilization method (IVF or ICSI). Using the DFI < 15% group as the reference, odds ratios (ORs) and 95% confidence intervals (CIs) were calculated for pregnancy outcomes in the other DFI categories. The independent factors associated with low birth weight were further determined through multivariate stepwise regression analysis.

All statistical analyses were performed using SAS 9.4 (SAS Institute, USA). A two - tailed P value < 0.05 was considered statistically significant.

## Results

### Baseline characteristics and semen parameters across DFI subgroups

This study included 5271 couples, with 3313 couples in the low DFI group (DFI < 15%), 1585 couples in the medium DFI group (15% ≤ DFI < 30%), and 373 couples in the high DFI group (DFI ≥ 30%). **Table 1** presents the baseline and clinical characteristics of patients stratified by DFI values. In the low DFI group, both male and female partners were younger compared to the medium and high DFI groups (P<0.05). The high DFI group had a higher proportion of primary infertility, which may be partly attributed to an increased incidence of male factor infertility. Other factors, including BMI, duration of infertility, ultrasonographic findings, and basal hormone levels, showed no statistically significant differences across the groups (P>0.05).

**Table 1:** Patient characteristics and male semen quality grouped by sperm DFI.

	DFI<15%	15%≤DFI<30%	DFI≥30%	P-value
Female				
Age (years)	30.91 ± 4.39	31.33 ± 4.44	31.37 ± 4.33	0.002
Body mass index (Kg/m^2^)	22.24 ± 3.22	22.35 ± 3.35	22.3 ± 3.06	0.783
Infertility duration (years)	3.62 ± 2.81	3.58 ± 2.71	3.68 ± 2.72	0.757
Pattern of infertility, n (%)				0.007
Primary	1269 (38.3)	632 (39.9)	174 (46.7)	
Secondary	2044 (61.7)	953 (60.1)	199 (53.4)	
Male factor, n (%)	481 (14.5)	553 (34.9)	212 (56.8)	<0.001
Ultrasonographic findings				
Antral follicle count (n)	14.47 ± 6.61	14.53 ± 6.27	13.89 ± 6.3	0.173
Endometrial thickness (mm)	1.78 ± 2.55	1.88 ± 2.66	1.69 ± 2.41	0.517
Intrauterine adhesions	772 (23.3)	347 (21.9)	72 (19.3)	0.157
Scar uterus, n (%)	454 (13.7)	204 (12.9)	57 (15.3)	0.440
Basal endocrine profile				
AMH (ng/mL)	3.8 ± 3.08	3.79 ± 3.07	3.63 ± 2.84	0.392
FSH (IU/mL)	6.43 ± 2.51	6.54 ± 2.74	6.52 ± 3.15	0.520
E2 (pg/mL)	50.68 ± 47.84	50.87 ± 49.11	54.09 ± 51.72	0.175
LH (mIU/mL)	6.18 ± 5.47	6.28 ± 5.36	6.75 ± 7.03	0.637
Male				
Age (years)	32.59 ± 4.86	33.35 ± 5.29	33.77 ± 5.37	<0.001
Body mass index (Kg/m^2^)	24.56 ± 4.33	25.91 ± 62.16	24.49 ± 4.34	0.130
Infertility duration (years)	3.34 ± 2.46	3.39 ± 2.45	3.35 ± 2.26	0.572
Pattern of infertility, n (%)				0.032
Primary	1652 (49.9)	816 (51.5)	212 (56.8)	
Secondary	1661 (50.1)	769 (48.5)	161 (43.2)	
FSH (IU/mL)	4.36 ± 2.33	4.67 ± 2.67	5.07 ± 4.13	<0.001
Semen parameter				
Sperm conc (×10^6^/mL)				<0.001
<15	211 (6.4)	212 (13.4)	72 (19.3)	
≥15	3102 (93.6)	1373 (86.6)	301 (80.7)	
PR (%)				<0.001
<32%	796 (24)	819 (51.7)	256 (68.6)	
≥32%	2517 (76)	766 (48.3)	117 (31.4)	
Normal morphology (%)				<0.001
<4%	173 (5.2)	203 (12.8)	76 (20.4)	
≥4%	3140 (94.8)	1382 (87.2)	297 (79.6)	
DFI	9.22 ± 3.34	20.17 ± 3.98	39.59 ± 9.87	<0.001

Data are presented as mean ± standard deviation or number (percentage).

AMH, Anti-Müllerian Hormone; FSH, follicle-stimulating hormone; E2, estrogen; LH, luteinizing hormone; PR, progressive motility spermatozoa.

Sperm DFI was negatively correlated with progressive motility, sperm concentration, and the percentage of normal morphology. Additionally, male FSH levels were significantly elevated in the high DFI group. No significant differences were observed in male BMI or infertility duration among the three groups.

### Cycle characteristics and laboratory outcomes

Cycle characteristics and laboratory outcomes grouped by the value of DFI are summarized in **Table 2**. No significant differences were observed in stimulation parameters across the three DFI groups (P>0.05). Regarding laboratory outcomes, compared to the low and medium DFI groups, the high DFI group had a lower proportion of IVF fertilization methods used (75.5%, 63.7%, 44.2%, respectively; P<0.001); however, there was no significant difference in the 2PN fertilization rate (64.83%, 61.25%, 61.29%, respectively; P=0.336) among the groups after IVF. The ICSI fertilization rate (80.94%, 79.92%, 76.26%, respectively; P<0.001), blastocyst formation rate (56.44%, 55.32%, 53.725%, respectively; P=0.045), and transplantable embryo rate (3.97 ± 2.71, 3.9 ± 2.70, 3.38 ± 2.40, respectively; P<0.001) were significantly lower in the high DFI group compared to the medium and low DFI groups (P<0.05).

**Table 2:** Cycle characteristics and laboratory outcomes grouped by sperm DFI.

	DFI<15%	15%≤DFI<30%	DFI≥30%	P-value
Cycle Characteristics				
Initial dose of Gn (IU)	168.94 ± 72.55	165.61 ± 62.97	168.03 ± 60.84	0.602
Total dose of Gn (IU)	2019.79 ± 789.79	2022.72 ± 846.2	2023.51 ± 724	0.768
uFSH/rFSH	1685.14 ± 568.27	1668.52 ± 554.94	1660.89 ± 494.33	0.765
HMG (U)	329.42 ± 558.95	342.06 ± 668.87	357.47 ± 592.38	0.271
Duration of Gn used (d)	10.57 ± 2.17	10.56 ± 2.32	10.5 ± 2.11	0.819
HCG trigger day				
Endometrial thickness (mm)	10.93 ± 2.58	10.99 ± 2.64	10.75 ± 2.68	0.161
E2 (pg/mL)	2057.91 ± 1210.74	2080.83 ± 1225.84	1996.64 ± 1260.9	0.144
LH (mIU/mL)	1.95 ± 1.63	2.02 ± 1.84	2.03 ± 1.68	0.128
P (ng/mL)	0.52 ± 0.33	0.52 ± 0.36	0.53 ± 0.32	0.529
No. of oocytes retrieved	12.95 ± 6.74	13.19 ± 7.07	12.52 ± 6.85	0.272
Laboratory Outcomes				
Fertilization method, n (%)				<0.001
Not fertilized	54 (1.6)	32 (2)	9 (2.4)	
IVF	2501 (75.5)	1010 (63.7)	165 (44.2)	
ICSI	631 (19.1)	456 (28.8)	179 (48)	
IVF+ICSI	127 (3.8)	87 (5.5)	20 (5.4)	
MII oocytes (ICSI), n (%)	5977/8439 (70.83)	4394/6163 (71.3)	1588/2292 (69.28)	0.194
2PN Fertility, n (%)	25848 (64.83)	12344 (65.67)	2593 (67.51)	0.001
IVF	20009 (61.96)	8093 (61.25)	1230 (61.29)	0.336
ICSI	4853 (80.94)	3522 (79.92)	1211 (76.26)	<0.001
Cleavage rate, n (%)	24928 (96.44)	11879 (96.23)	2478 (95.56)	0.064
High quality embryo rate, n (%)	6412 (25.72)	2973 (25.03)	598 (24.13)	0.115
Blastocysts formed, n (%)	9660 (56.44)	4469 (55.32)	866 (53.72)	0.045
Number of transferable embryos, n	3.97 ± 2.71	3.90 ± 2.70	3.38 ± 2.40	<0.001
Cancellation rate, n (%)	212 (6.4)	92 (5.8)	26 (7)	0.609
Fresh embryo transfer rate, n (%)	2066/3101 (66.6)	1010/1493 (67.7)	227/347 (65.4)	0.662
Number of fresh embryos transferred, n (%)				0.094
1	1023/2066 (49.5)	458/2066 (45.4)	109/2066 (48)	
2	1043/2066 (50.5)	552/2066 (54.7)	118/2066 (52)	
Stage of fresh embryos transferred, n (%)				0.016
Cleavage	1254/2066 (60.7)	658/2066 (65.2)	153/2066 (67.4)	
Blastocyst	812/2066 (39.3)	352/2066 (34.9)	74/2066 (32.6)	

Data are presented as mean ± standard deviation or number (percentage).

Gn, gonadotropin; HMG, human menopausal gonadotropin; HCG, human chorionic gonadotropin; P, progesterone; IVF, *in vitro* fertilization; ICSI, intracytoplasmic sperm injection; MII, metaphase II; 2PN, two pronuclei.

In fresh embryo transfer, the number of embryos transferred did not differ significantly among the three groups (P>0.05). However, the proportion of cleavage-stage embryo transfers was significantly higher in the high DFI group compared to the other groups (P<0.05). These findings are detailed in **Table 2**.

### Clinical outcomes across DFI groups

**Table 3** presents the pregnancy outcomes following fresh embryo transfer across the three DFI groups. No significant differences were observed in the positive hCG rate (76.6%, 75,3%, 74.9%, respectively; P=0.659), clinical pregnancy rate (68.1%, 67.3%, 66.5%, respectively; P= 0.849), multiple birth rate (20.1%, 21.5%, 20.8%, respectively; P=0.228), miscarriage rate (12.9%, 11.9%, 10.6%, respectively; P=0.616), or live birth rate (58.9%, 58.4%, 59.0%, respectively; P= 0.969) among the groups. These results remained consistent in both crude and adjusted analyses.

**Table 3:** Clinical outcomes and single/multiple factor analysis of fresh embryo transfer grouped by sperm DFI.

	DFI<15%	15%≤DFI<30%	DFI≥30%	P-value
Positive HCG, n (%)	1582/2066 (76.6)	760/1010 (75.3)	170/227 (74.9)	0.659
Crude OR (95%CI)	REF	0.93 (0.78-1.11)	0.91 (0.67-1.25)	
Adjusted OR (95%CI)	REF	0.95 (0.79-1.15)	1.02 (0.72-1.44)	
Biochemical abortion rate, n (%)	176/1582 (11.1)	80/760 (10.5)	19/170 (11.2)	0.905
Crude OR (95%CI)	REF	0.94 (0.71-1.24)	1.01 (0.61-1.66)	
Adjusted OR (95%CI)	REF	0.9 (0.67-1.22)	0.88 (0.51-1.53)	
Clinical pregnancy, n (%)	1406/2066 (68.1)	680/1010 (67.3)	151/227 (66.5)	0.849
Crude OR (95%CI)	REF	0.97 (0.82-1.14)	0.93 (0.7-1.25)	
Adjusted OR (95%CI)	REF	1 (0.84-1.19)	1.07 (0.78-1.47)	
Multiple birth rate, n (%)	282/1406 (20.1)	146/680 (21.5)	39/151 (25.8)	0.228
Crude OR (95%CI)	REF	1.09 (0.87-1.36)	1.39 (0.94-2.04)	
Adjusted OR (95%CI)	REF	0.95 (0.79-1.15)	1.02 (0.72-1.44)	
Miscarriage, n (%)	182/1406 (12.9)	81/680 (11.9)	16/151 (10.6)	0.616
Crude OR (95%CI)	REF	0.91 (0.69-1.2)	0.8 (0.46-1.37)	
Adjusted OR (95%CI)	REF	0.79 (0.58-1.06)	0.58 (0.32-1.06)	
Live birth, n (%)	1216/2066 (58.9)	590/1010 (58.4)	134/227 (59)	0.969
Crude OR (95%CI)	REF	0.98 (0.84-1.14)	1.01 (0.76-1.33)	
Adjusted OR (95%CI)	REF	1.05 (0.89-1.24)	1.19 (0.88-1.62)	

Adjusted factors: age of both male and female partners, type of infertility, cause of infertility (excluding male factor infertility), number of transplanted embryos, and type of transplanted embryos.

REF, Reference; CI, confidence interval; OR, odds ratio.

### Maternal and neonatal outcomes in singleton pregnancies

Among the 5271 couples included in the study, 1570 couples achieved a singleton live birth. The maternal and neonatal outcomes were analyzed across the three DFI groups (**Table 4**). Overall, no significant differences were observed in most outcomes among the groups (P>0.05). However, in the high DFI group, the proportion of low birth weight (LBW) newborns was significantly higher compared to the low and medium DFI groups (3.9%, 6.6%, 10.1%, respectively; P=0.006). In further stepwise multivariate regression analysis (**Table 5**), 15% ≤ DFI < 30% and DFI ≥ 30% were consistently associated with neonatal low birth weight (OR: 1.75, 95% CI 1.08–2.85, P= 0.023; OR: 2.78, 95% CI 1.34–5.75, P=0.006, respectively). Additionally, neonatal gender (OR: 1.67, 95% CI 1.06–2.62, P=0.028) and gestational age (OR: 0.33, 95% CI 0.27–0.39, P<0.001) were also significant risk factors for low birth weight.

**Table 4:** Obstetric and perinatal outcomes in singleton pregnancies of couples grouped by sperm DFI.

	DFI<15%	15%≤DFI<30%	DFI≥30%	P-value
Total live birth, n	1003	468	99	
Maternal Outcomes				
HDP, n (%)	32 (3.2)	15 (3.2)	2 (2)	0.809
GDM, n (%)	138 (13.8)	78 (16.7)	9 (9.1)	0.102
ICP, n (%)	4 (0.4)	2 (0.4)	0 (0)	1.000
Abnormal placentation, n (%)	54 (5.4)	25 (5.3)	8 (8.1)	0.521
Placenta previa, n (%)	28 (2.8)	11 (2.4)	4 (4)	0.636
PROM, n (%)	23 (2.3)	16 (3.4)	3 (3)	0.448
Postpartum hemorrhage, n (%)	2 (0.2)	0 (0)	0 (0)	1.000
Cesarean delivery, n (%)	594 (59.2)	270 (57.7)	61 (61.6)	0.731
Neonatal Outcomes				
Gender, n (%)				0.564
Male	557 (55.5)	247 (52.8)	52 (52.5)	
Female	446 (44.5)	221 (47.2)	47 (47.5)	
Gestational age (weeks), n (%)	38.65 ± 1.57	38.62 ± 1.71	38.54 ± 1.74	0.972
Preterm birth	97 (9.7)	40 (8.6)	13 (13.1)	0.362
Very preterm birth	11 (1.1)	4 (0.9)	0 (0)	0.755
Postterm birth	0 (0)	0 (0)	0 (0)	1.000
Birthweight (g), n (%)	3251.65 ± 476.44	3228.49 ± 523.82	3155.56 ± 493.51	0.435
Low birthweight	39 (3.9)	31 (6.6)	10 (10.1)	0.006
Very low birthweight	4 (0.4)	3 (0.6)	1 (1)	0.377
Macrosomia	41 (4.1)	18 (3.9)	2 (2)	0.596
Z-score, n (%)	0.22 ± 1.03	0.17 ± 1.03	0.03 ± 0.91	0.373
Small - for - gestational age	52 (5.2)	35 (7.5)	7 (7.1)	0.202
Large - for - gestational age	129 (12.9)	67 (14.3)	8 (8.1)	0.240
Major birth defects, n (%)	19 (1.9)	13 (2.8)	0 (0)	0.179

Data are presented as mean ± standard deviation or number (percentage).

HDP, Hypertensive disorders in pregnancy; ICP, Intrahepatic cholestasis of pregnancy; GDM, Gestational diabetes mellitus; PROM, premature rupture of membranes.

**Table 5:** Stepwise logistic regression on risk of low birth weight.

Factor	Category	Crude OR (95%CI)	P-value	Adjusted OR (95%CI)	P-value
DFI	< 15%	REF		REF	
	15% - 30%	1.75 (1.08-2.85)	0.0232	2.74 (1.34-5.59)	0.006
	≥ 30%	2.78 (1.34-5.75)	0.0059	3.56 (1.19-10.69)	0.024
Gender	Male	REF		REF	
	Female	1.67 (1.06-2.62)	0.0281	4.73 (2.26-9.91)	<.001
Gestational age (continuous variable)		0.33 (0.27-0.39)	<.0001	0.29 (0.24-0.36)	<.001

REF, Reference; CI, confidence interval; OR, odds ratio.

## Discussion

Based on DFI values, our retrospective analysis demonstrated that patients with high DFI exhibited a significantly lower sperm quality. In addition, high DFI was found to impact embryonic development and increase the risk of low birth weight in offspring. However, no significant effects of DFI were observed on pregnancy, obstetrical or other neonatal outcomes.

Several studies have recommended incorporating sperm DNA fragmentation analysis as a routine complementary test in semen evaluation (6, 15, 22). In this study, we utilized the SCSA method due to its high sensitivity in detecting sperm DNA fragmentation (23, 24). Our findings reveal that high DFI is strongly associated with significant impairments in all seminal parameters. Additionally, couples with high DFI are more likely to seek assisted reproduction due to male factors, with a higher proportion opting for ICSI fertilization in subsequent treatments. These results are consistent with the majority of published studies (24–26), which emphasize that sperm DNA fragmentation—characterized by breaks or damage in the genetic material of spermatozoa - is a key contributor to male infertility. Such damage compromises the functional capacity of sperm, leading to difficulties in achieving natural conception or optimal outcomes in assisted reproduction.

Regarding the impact of DFI on embryonic development and clinical outcomes, our study found the fertilization rate in the ICSI group was negatively correlated with DFI values, whereas no significant differences were observed among the IVF groups. During ICSI procedure, sperm are subjectively selected based on morphological and motility criteria by operators, with little consideration for DNA integrity. This may explain the lower fertilization rates and poorer embryo quality observed with higher DFI (27, 28). In contrast, the IVF procedure involves a natural sperm selection process, potentially mitigating the impact of high DFI on fertilization outcomes. When observing the development of embryos, our findings is consistent with most published studies (29, 30), indicating that high DFI negatively affects blastocyst formation and the rate of available embryos. Given our center’s advocacy for single blastocyst transfer, fresh embryo transfer statistics revealed that patients with high DFI were more likely to undergo cleavage embryo transfer. This trend may stem from the reduced blastocyst formation rates and developmental arrest of embryos associated with high DFI (31–34). Recent studies have shown that Physiological Intracytoplasmic Sperm Injection (PICSI) can significantly improve both fertilization rates and embryo quality (35, 36), when hyaluronic acid bound sperm selection method is used, it generally selects a more genomic mature sperm with intact DNA, which can enhance embryological outcome (36). However, our center currently does not offer PICSI, and therefore, we are currently unable to implement such measures and make comparisons. This remains a promising area for future exploration among men with high DFI.

There is ongoing controversy regarding the impact of DFI on clinical outcomes, particularly its influence on miscarriage risk (30, 37). Our results indicate no significant differences among the three groups in terms of biochemical pregnancy rate, clinical pregnancy rate, miscarriage rate, multiple birth rate, or live birth rate. This may be attributed to the repair capacity of oocytes and early embryos, which can mitigate sperm DNA damage to a certain extent (38, 39). However, this repair mechanism is limited, and beyond a certain threshold, insufficient or aberrant repair may lead to mutations in the zygote genome, potentially resulting in pathologies in the offspring (40). In the present study, there was no significant difference in most obstetric and neonatal complications among the three groups. However, the proportion of low birth weight infants increased with higher DFI values, and this finding remained statistically significant even after adjusting for potential confounders. These results are consistent with Li et al.’s study (41). We further performed stepwise logistic regression analysis for risk of low birth weight, revealing that DFI, gestational age, and neonatal gender were all independent factors. However, there are some residual confounding factors, such as maternal nutrition and weight gain during pregnancy, that were not included in the study. Therefore, further prospective research is still needed to confirm our finding.

There are several limitations to this study that should be acknowledged. First, the retrospective design may have introduced bias. While the inclusion of a large dataset enabled us to account for critical confounding factors, our findings could still be influenced by some unmeasured confounders. Second, the lack of data linkage to electronic medical records may result in the underreport of maternal and neonatal outcomes, which may have influenced the statistical power. Lastly, due to time constraints, we were unable to complete live birth follow-up for the entire cohort; Lastly, due to time constraints, we were unable to complete live birth follow-up for the entire cohort. Furthermore, our analysis was limited to pregnancy outcomes following fresh embryo transfer, leaving the potential effects of frozen-thawed embryo transfers unexplored, warranting further investigation.

## Conclusion

Our study demonstrates that high sperm DFI is significantly associated with impairments in all semen parameters. Elevated DFI negatively impacts fertilization, the formation of usable blastocysts, and the rate of available embryos. While high DFI may contribute to an increased incidence of low birth weight in offspring, it does not significantly impact key clinical outcomes or other maternal and neonatal complications. Continued surveillance of offspring conceived through assisted reproduction in such cases is essential to monitor potential long-term health effects.

## Data Availability

The original contributions presented in the study are included in the article/Supplementary Material. Further inquiries can be directed to the corresponding authors.

## References

[1] Mélodie VB Christine W. Fertility and infertility: Definition and epidemiology. Clin Biochem. (2018) 62:2–10. doi: 10.1016/j.clinbiochem.2018.03.012 29555319

[2] Rotondo JC Lanzillotti C Mazziotta C Tognon M Martini F. Epigenetics of male infertility: the role of DNA methylation. Front Cell Dev Biol. (2021) 9:689624. doi: 10.3389/fcell.2021.689624 34368137 PMC8339558

[3] Lewis SEM. Is sperm evaluation useful in predicting human fertility? Reproduction. (2007) 134:31–40. doi: 10.1530/REP-07-0152 17641086

[4] Simon L Castillo J Oliva R Lewis SEM. Relationships between human sperm protamines, DNA damage and assisted reproduction outcomes. Reprod BioMed Online. (2011) 23:724–34. doi: 10.1016/j.rbmo.2011.08.010 22036908

[5] Alkhayal A Gabriel San M Zeidan K Alrabeeah K Noel D McGraw R. Sperm DNA and chromatin integrity in semen samples used for intrauterine insemination. J Assist Reprod Genet. (2013) 30:1519–24. doi: 10.1007/s10815-013-0101-3 PMC3879931 24068511

[6] Agarwal A Said TM. Role of sperm chromatin abnormalities and DNA damage in male infertility. Hum Reprod Update. (2003) 9:331–45. doi: 10.1093/humupd/dmg027 12926527

[7] Zini A. Are sperm chromatin and DNA defects relevant in the clinic? Syst Biol Reprod Med. (2011) 57:78–85. doi: 10.3109/19396368.2010.515704 21208147

[8] Simon L Emery BR Carrell DT. Review: Diagnosis and impact of sperm DNA alterations in assisted reproduction. Best Pract Res Clin Obstet Gynaecol. (2017) 44:38–56. doi: 10.1016/j.bpobgyn.2017.07.003 28935366

[9] Donald E Regina W. Meta-analysis of sperm DNA fragmentation using the sperm chromatin structure assay. Reprod BioMed Online. (2006) 12:466–72. doi: 10.1016/s1472-6483(10)62000-7 16740220

[10] McQueen DB Zhang J Robins JC. Sperm DNA fragmentation and recurrent pregnancy loss: a systematic review and meta-analysis. Fertil Steril. (2019) 112(1):54–60 e3. 10.1016/j.fertnstert.2019.03.003 31056315

[11] Gat I Tang K Quach K Kuznyetsov V Antes R Filice M. Sperm DNA fragmentation index does not correlate with blastocyst aneuploidy or morphological grading. PloS one. (2017) 12(6):e0179002. 28591199 10.1371/journal.pone.0179002 PMC5462460

[12] Cissen M Wely M Scholten I Mansell S Bruin J Mol B. Measuring sperm DNA fragmentation and clinical outcomes of medically assisted reproduction: A systematic review and meta-analysis. PloS One. (2016) 11:e0165125. doi: 10.1371/journal.pone.0165125 27832085 PMC5104467

[13] Basar M Kahraman S. Clinical utility of sperm DNA fragmentation testing: practice recommendations based on clinical scenarios. Trans Androl Urol. (2017) 6:S574–6. doi: 10.21037/tau.2017.04.40 PMC5643703 29082180

[14] Bender Atik R Christiansen OB Elson J Kolte AM Lewis S Middeldorp S. ESHRE guideline: recurrent pregnancy loss: an update in 2022. Hum Reprod Open. (2023) 2023:e13739. doi: 10.1093/hropen/hoad002 PMC9982362 36873081

[15] Lars B Jackson KB. The sixth edition of the WHO Laboratory Manual for the Examination and Processing of Human Semen: ensuring quality and standardization in basic examination of human ejaculates. Fertil Steril. (2022) 117:246–51. doi: 10.1016/j.fertnstert.2021.12.012 34986984

[16] Donald P E Kay K Regina L W. Analysis of sperm DNA fragmentation using flow cytometry and other techniques. Soc Reprod Fertil Suppl. (2007) 65:93–113. 17644957

[17] Devroey P Van Steirteghem A. A review of ten years experience of ICSI. Hum Reprod Update. (2004) 10:19–28. doi: 10.1093/humupd/dmh004 15005461

[18] Zheng D Zeng L Yang R Lian Y Zhu YM Liang X. Intracytoplasmic sperm injection (ICSI) versus conventional *in vitro* fertilisation (IVF) in couples with non-severe male infertility (NSMI-ICSI): protocol for a multicentre randomised controlled trial. BMJ Open. (2019) 9:e030366. doi: 10.1136/bmjopen-2019-030366 PMC6773417 31575574

[19] Maria José D Susanna A Giovanni C Sophie D Kersti L Carlos PE. Revised guidelines for good practice in IVF laboratories(2015). Hum Reprod. (2016) 31(4):685–6. 10.1093/humrep/dew016 26908842

[20] Cummins JM Breen TM Harrison KL Shaw JM Wilson LM Hennessey JF. A formula for scoring human embryo growth rates in *in vitro* fertilization: its value in predicting pregnancy and in comparison with visual estimates of embryo quality. J In Vitro Fert Embryo Transf. (1986) 3:284–95. doi: 10.1007/BF01133388 3783014

[21] Ahmadi A Ng S. Developmental capacity of damaged spermatozoa. Human Reprod (Oxford, England). (1999) 14(9):2279–85. 10.1093/humrep/14.9.2279 10469696

[22] Aitken RJ Bakos HW. Should we be measuring DNA damage in human spermatozoa? New light an old Quest Hum Reprod. (2021) 36:1175–85. doi: 10.1093/humrep/deab004 33532854

[23] Evenson D. Sperm chromatin structure assay (SCSA^®^). Methods Mol Biol (Clifton N.J.). (2013) 927:147–64. doi: 10.1007/978-1-62703-038-0_14 22992911

[24] Evenson D Djira G Kasperson K Christianson J. Relationships between the age of 25,445 men attending infertility clinics and sperm chromatin structure assay (SCSA^®^) defined sperm DNA and chromatin integrity. Fertil Steril. (2020) 114:311–20. doi: 10.1016/j.fertnstert.2020.03.028 32653083

[25] Campos LGA Requejo LC Minano CAR Orrego JD Loyaga EC Cornejo LG. Correlation between sperm DNA fragmentation index and semen parameters in 418 men seen at a fertility center. JBRA Assist Reprod. (2021) 25:349–57. doi: 10.5935/1518-0557.20200079 PMC8312297 33624489

[26] Stavros S Potiris A Molopodi E Mavrogianni D Zikopoulos A Louis K. Sperm DNA fragmentation: unraveling its imperative impact on male infertility based on recent evidence. Int J Mol Sci. (2024) 25. doi: 10.3390/ijms251810167 PMC11432134 39337652

[27] Tarozzi N Nadalini M Borini A. Effect on sperm DNA quality following sperm selection for ART: new insights. Genet Damage Hum Spermatozoa. (2019), 169–87. doi: 10.1007/978-3-030-21664-1_10 31301052

[28] Asgari F Nadalini M Borini A. Risk of embryo aneuploidy is affected by the increase in sperm DNA damage in recurrent implantation failure patients under ICSI-CGH array cycles. Hum Fertil. (2021) 25:872–80. doi: 10.1080/14647273.2021.1920054 33938375

[29] Green KA Patounakis G Dougherty MP Werner MD Scott RT Franasiak JM. Sperm DNA fragmentation on the day of fertilization is not associated with embryologic or clinical outcomes after IVF/ICSI. J Assisted Reprod Genet. (2019) 37:71–6. doi: 10.1007/s10815-019-01632-5 PMC7000566 31755002

[30] Maghraby H Elsuity MA Adel N Magdi Y Abdelbadie AS Rashwan MM. Quantifying the association of sperm DNA fragmentation with assisted reproductive technology outcomes: An umbrella review. BJOG: Int J Obstet Gynaecol. (2024) 131:1181–96. doi: 10.1111/1471-0528.17796 38450853

[31] Newman H Catt S Vining B Vollenhoven B Horta F. DNA repair and response to sperm DNA damage in oocytes and embryos, and the potential consequences in ART: a systematic review. Mol Hum Reprod. (2022) 28. doi: 10.1093/molehr/gaab071 34954800

[32] Ménézo Y Dale B Cohen M. DNA damage and repair in human oocytes and embryos: a review. Zygote (Cambridge England). (2010) 18:357–65. doi: 10.1017/S0967199410000286 20663262

[33] Musson R Gąsior Ł Bisogno S Ptak G. DNA damage in preimplantation embryos and gametes: specification, clinical relevance and repair strategies. Hum Reprod Update. (2022) 28:376–99. doi: 10.1093/humupd/dmab046 PMC9071077 35021196

[34] Chapuis A Gala A Ferrieres-Hoa A Mullet T Bringer-Deutsch S Vintejoux E. Sperm quality and paternal age: effect on blastocyst formation and pregnancy rates. Basic Clin Androl. (2017) 27:2. doi: 10.1186/s12610-016-0045-4 28127436 PMC5251225

[35] West R Coomarasamy A Frew L Hutton R Kirkman-Brown J Lawlor M. Sperm selection with hyaluronic acid improved live birth outcomes among older couples and was connected to sperm DNA quality, potentially affecting all treatment outcomes. Hum Reprod. (2022) 37:1106–25. doi: 10.1093/humrep/deac058 PMC9156852 35459947

[36] Shivhare S Karunakaran S Bose AS Goel R Ananthakrishnan R. Does physiological intracytoplasmic sperm injection improve outcome in men with abnormal semen parameters: A retrospective cohort study. J Hum Reprod Sci. (2024) 17:200–6. doi: 10.4103/jhrs.jhrs_95_24 PMC11559347 39544679

[37] Maria Christine K Reinhardt Josefine N Anna S Vauvert Kathrine H Marie Astrid K David W. Prospective reproductive outcomes according to sperm parameters, including DNA fragmentation, in recurrent pregnancy loss. Reprod BioMed Online. (2024) 49(2):103773. doi: 10.1016/j.rbmo.2023.103773 38879918

[38] Matsuda Y Tobari I. Chromosomal analysis in mouse eggs fertilized *in vitro* with sperm exposed to ultraviolet light (UV) and methyl and ethyl methanesulfonate (MMS and EMS). Mutat Res. (1988) 198:131–44. doi: 10.1016/0027-5107(88)90048-6 3352623

[39] Martin J Aitken R Bromfield E Nixon B. DNA damage and repair in the female germline: contributions to ART. Hum Reprod Update. (2019) 25:180–201. doi: 10.1093/humupd/dmy040 30541031

[40] Saleh R Agarwal A Nada E El-Tonsy M Sharma R Meyer A. Negative effects of increased sperm DNA damage in relation to seminal oxidative stress in men with idiopathic and male factor infertility. Fertil Steril. (2003) p:1597–605. doi: 10.1016/S0015-0282(03)00337-6 12801566

[41] Li F Duan X Li M Ma X. Sperm DNA fragmentation index affect pregnancy outcomes and offspring safety in assisted reproductive technology. Sci Rep. (2024) 14. doi: 10.1038/s41598-023-45091-6 PMC10764900 38172506

